# A CT-based radiomics nomogram for predicting histologic grade and outcome in chondrosarcoma

**DOI:** 10.1186/s40644-024-00695-7

**Published:** 2024-04-11

**Authors:** Xiaoli Li, Xianglong Shi, Yanmei Wang, Jing Pang, Xia Zhao, Yuchao Xu, Qiyuan Li, Ning Wang, Feng Duan, Pei Nie

**Affiliations:** 1 Department of Radiology, The Affiliated Hospital of Qingdao University, No. 369, Shanghai Road, 266000 Qingdao, Qingdao, Shandong China; 2 GE Healthcare China, Pudong New Town, Shanghai, China; 3 Department of Radiology, The Affiliated Hospital of Shandong University of Traditional Chinese Medicine, Jinan, Shandong China; 4 School of Nuclear Science and Technology, University of South China, Hengyang, Hunan China; 5 Department of Radiology, Shandong Provincial Hospital Affiliated to Shandong First Medical University, No. 324, Jingwu Road, 250021 Jinan, Shandong China

**Keywords:** Chondrosarcoma, Heterogeneity, Outcome, Radiomics, Computed tomography

## Abstract

**Objective:**

The preoperative identification of tumor grade in chondrosarcoma (CS) is crucial for devising effective treatment strategies and predicting outcomes. The study aims to build and validate a CT-based radiomics nomogram (RN) for the preoperative identification of tumor grade in CS, and to evaluate the correlation between the RN-predicted tumor grade and postoperative outcome.

**Methods:**

A total of 196 patients (139 in the training cohort and 57 in the external validation cohort) were derived from three different centers. A clinical model, radiomics signature (RS) and RN (which combines significant clinical factors and RS) were developed and validated to assess their ability to distinguish low-grade from high-grade CS with area under the curve (AUC). Additionally, Kaplan-Meier survival analysis was applied to examine the association between RN-predicted tumor grade and recurrence-free survival (RFS) of CS. The predictive accuracy of the RN was evaluated using Harrell’s concordance index (C-index), hazard ratio (HR) and AUC.

**Results:**

Size, endosteal scalloping and active periostitis were selected to build the clinical model. Three radiomics features, based on CT images, were selected to construct the RS. Both the RN (AUC, 0.842) and RS (AUC, 0.835) were superior to the clinical model (AUC, 0.776) in the validation set (*P* = 0.003, 0.040, respectively). A correlation between Nomogram score (Nomo-score, derived from RN) and RFS was observed through Kaplan-Meier survival analysis in the training and test cohorts (log-rank *P* < 0.050). Patients with high Nomo-score tumors were 2.669 times more likely to suffer recurrence than those with low Nomo-score tumors (HR, 2.669, *P* < 0.001).

**Conclusions:**

The CT-based RN performed well in predicting both the histologic grade and outcome of CS.

**Supplementary Information:**

The online version contains supplementary material available at 10.1186/s40644-024-00695-7.

## Introduction

Chondrosarcoma (CS) is the third most common malignant bone tumor, comprising approximately 20–27% of all primary malignant bone tumors [1]. According to the 2020 update of the World Health Organization classification of primary musculoskeletal soft-tissue and bone tumors, CS is classified into low-grade and high-grade based on the cellularity, vascularization, nuclear atypia and muco-myxoid matrix of the tumor [2, 3]. Low-grade CSs (grade I/atypical cartilaginous tumor [ACT]) exhibit local aggressiveness and infrequent metastasis. Typically, these conditions are managed through watchful waiting or surgical intervention (curettage) [4]. The overall 10-year survival rate stands at 88% [5]. Conversely, high-grade (grade II and III) commonly involve metastasis and are fatal for most patients. The 10-year survival rate drops significantly to only 26% [5]. Therefore, it becomes crucial to preoperatively differentiate between low-grade and high-grade CS, as it would facilitate precise individualized management and ultimately improve patient prognosis.

CT has emerged as an important imaging modality for the preoperative assessment of CS. Numerous studies have aimed to differentiate low-grade from high-grade CS using both qualitative and quantitative methods [6–8]. However, the effectiveness of traditional CT features in grading tumors is limited. This is because low-grade and high-grade CS share similar radiological features, such as deep endosteal scalloping, calcification, cortical destruction, and soft tissue masses [6]. Additionally, Del Grande F et al. found no statistically significant difference in mean tumor size between grade I CS (56 mm) and grade II CS (57 mm) [9]. Consequently, a preferred imaging-based modality, such as radiomics, is crucial to develop and provide more reliable preoperative characteristics for differentiating between low-grade and high-grade CS.

Radiomics is an innovative imaging-based instrument that converts medical images into quantitative high-dimensional data, providing a comprehensive analysis of tumor heterogeneity throughout the entire tumor volume. This approach surpasses the limitations of conventional imaging modalities and sampling biopsies by allowing a detailed non-invasive assessment of tumor characteristics. Recent studies have demonstrated the promising performance of radiomics in terms of differentiation, tumor staging and outcome prediction of bone tumors [10–12]. Several studies have also explored the value of MR/CT-based radiomics in differentiating CS from other cartilaginous tumors and estimating early recurrence in pelvic CS [13–15]. However, there is currently limited research focusing on the use of radiomics for predicting the histologic grade of CS and investigating the association between RN-predicted tumor grade and prognosis in CS.

In this study, we constructed and validated a CT-based radiomics nomogram (RN) for differentiating low-grade from high-grade CS. Furthermore, the correlation between the RN-predicted tumor grade and the prognosis of CS patients was evaluated.

## Materials and methods

### Patient selection

**Fig. 1:**
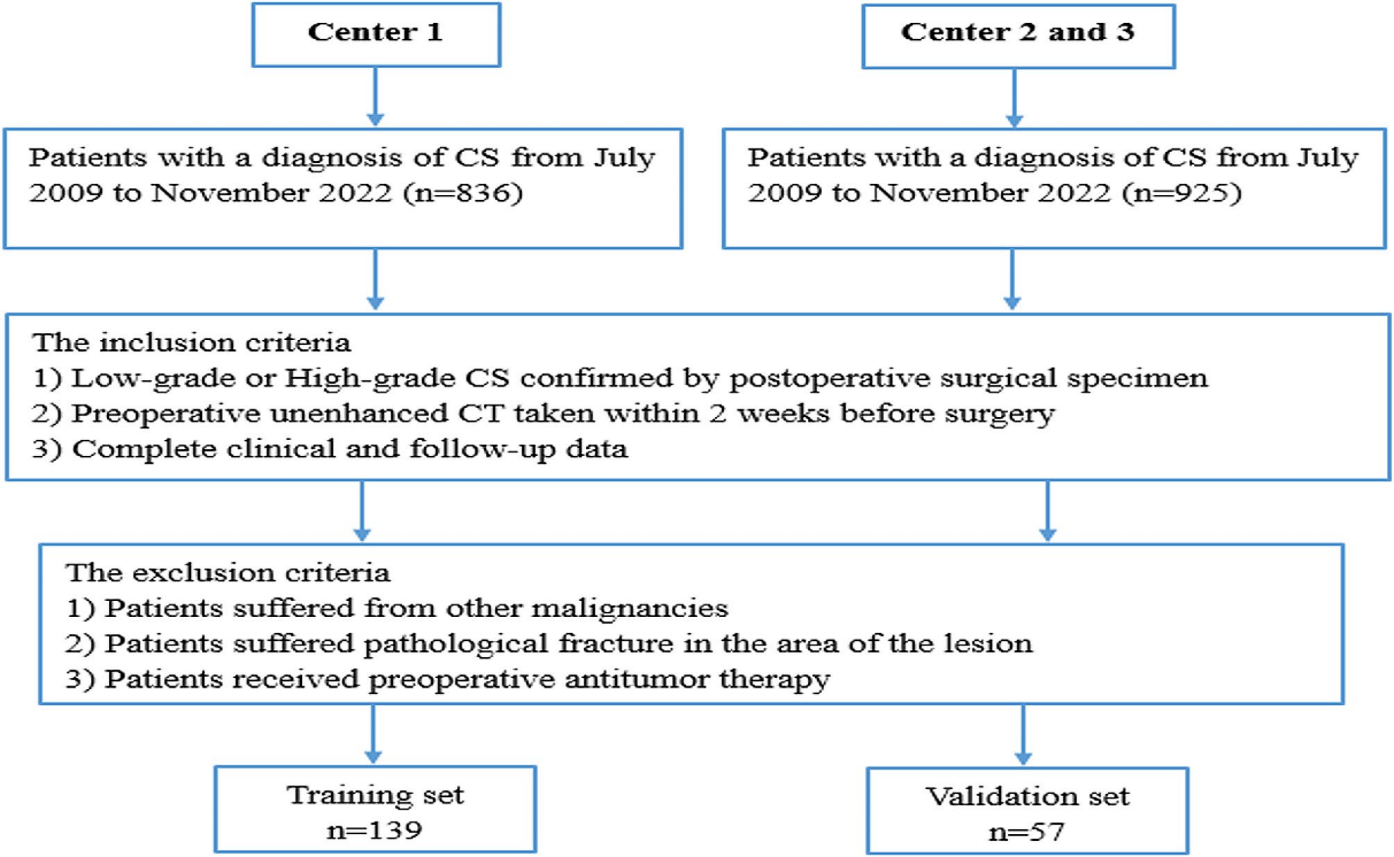
The flow diagram of the study population

**Fig. 2:**
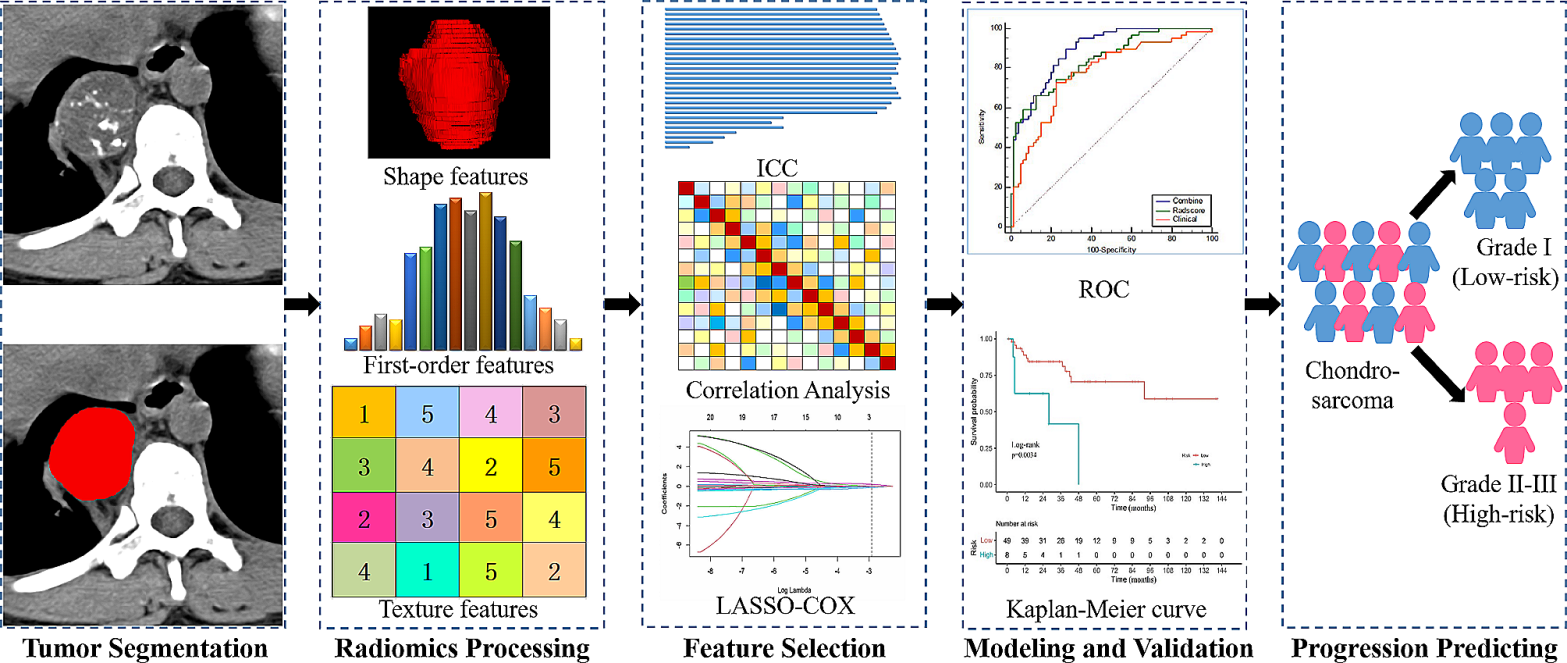
The workflow of the multicenter study

This retrospective study was approved by the Institutional Review Board of three centers (Centers 1, 2 and 3) from July 2009 to November 2022. The requirement for informed consent was waived. The inclusion criteria were as follows: (i) definitive histological diagnosis of CS confirmed by surgical specimen assessment; (ii) unenhanced CT scan taken within 2 weeks before surgery; and (iii) complete clinical and follow-up data. We excluded patients if they suffered from other malignancies, or pathological fracture in the area of the lesion, or received administration of preoperative antitumor therapy. Finally, 139 CS patients from Center 1 constituted the training cohort, whereas 57 CS patients constituted the external test cohort from Center 2 and 3. The patient recruitment pathway is shown in Fig. 1. The workflow is shown in Fig. 2.

Baseline clinical factors included patients’ demographics (gender, age, pain) and conventional radiologic factors. All data were retrieved through the electronic medical records. The conventional imaging characteristics were as follows [16]: tumor size (the maximum tumor length), tumor location (long bone/non-long bone), and tumor type (central type/peripheral type), depth of endosteal scalloping (deep/not deep; deep endosteal scalloping was defined as cortical thinning of more than two-thirds of the cortical thickness [17]), entrapped fat (present or absent), and calcification (present or absent), bone expansion (present or absent), and cortical destruction (present or absent), active periostitis (present or absent) and soft tissue mass (present or absent).

### CT acquisition, tumor segmentation and radiomics feature extraction

The CT scan parameters and three-dimensional (3-D) segmentation of the region of interest (ROI) are shown in Supplementary Methods and Fig. 2. To standardize the CT images, image resampling, gray-level normalization and discretization were employed before feature extraction. Four types of radiomics features, including 18 intensity statistic features, 14 shape features, 93 texture features, and 1284 filter and wavelet features, were extracted using the Radcloud platform (Huiying Medical Technology Co., Ltd) [18]. In addition, fifteen filters were applied to the original images to derive specific images for each patient, including exponential, logarithm, square, square root, gradient, local binary patterns-two-dimension, local binary patterns-three-dimension-k, and wavelets [low-high-low (LHL), low-high-high (LHH), high-low-low (HLL), low-low-high (LLH), high-low-high (HLH), high-high-high (HHH), high-high-low (HHL), and low-low-low (LLL)]. A total of 1409 radiomics features were extracted. The details are presented in Supplementary Methods.

Thirty cases were randomly selected and segmented by two Readers to assess the inter- and intra- class correlation coefficients (ICCs) [19]. Reader 2 repeated the ROI delineation twice within a 2-week period following the same procedure. Radiomics features were considered stable when the ICC value exceeded 0.750. The remaining segmentations were performed by Reader 1.

### Clinical model construction

In the training cohort, two steps were applied to select the significant clinical factors used for developing the clinical model, including univariate analysis and multivariate regression analysis. Odds ratios (ORs) and 95% confidence intervals (CIs) were obtained for each independent factor.

### Radiomics signature (RS) construction

Dimension reduction of the radiomics features was performed with three steps, including ICC, one-way analysis of variance (ANOVA), least absolute shrinkage and selection operator (LASSO) regression model. Ten-fold cross-validation via minimum criteria was used to select the tuning parameter (λ) in the LASSO regression model. The RS was developed by combining the most valuable factors with their nonzero corresponding coefficients. For each case, a radiomics score (Rad-score) was computed with the coefficients weighted by the LASSO regression model.

### RN construction and validation

Basing on the significant clinical variables and Rad-score, the RN was constructed. A radiomics-nomogram score (Nomo-score) was calculated. The performance of the three models (clinical model, RS, and RN) in differentiating low-grade from high-grade CS was evaluated using the area under the receiver operator characteristic (ROC) curve (AUC).

### Follow‑up and survival analysis

After surgery, the patients were followed up every 6–12 months during the first two years, and then annually. The date of the final follow-up was December 31, 2022. Recurrence-free survival (RFS) was calculated from the date of the surgery until either the date of recurrence, or the last negative follow-up or death. Follow-up data were attained through the medical records, including CT images and physical exams. Medical insurance records and telephone enquiries were also used.

Kaplan-Meier survival analysis was used to assess the association between the actual grade or RN-predicted grade and the RFS. The survival difference between the high-grade and low-grade groups was evaluated with the log-rank test. Harrell’s concordance index (C-index), hazard ratio (HR) and AUC were used for evaluating the predictive accuracy of the RN.

### Statistical analysis

Data analysis was conducted using R software (version 3.3.3; https://www.r-project.org) and SPSS (version 20.0; SPSS, Chicago, IL, USA). Univariate and multivariate regression analysis were conducted using the SPSS software. In addition, ICC, LASSO regression analysis, and survival analysis, C-index, and AUC were performed with the R software. A two-sided *P* value < 0.050 indicated statistical significance.

## Results

### Clinical model construction

Baseline clinical factors are shown in Table 1. A significant difference in age, size, and endosteal scalloping, soft mass, and active periostitis was found between low-grade and high-grade CS in the training cohort (*P* < 0.050). These five significant factors were input into multivariate regression analysis. Finally, size (*P* = 0.011; OR, 1.010; 95% CI, 1.000 ∼ 1.020), endosteal scalloping (*P* < 0.001; OR, 4.420; 95% CI, 2.360 ∼ 8.280) and active periostitis (*P* = 0.014; OR, 3.310; 95% CI, 1.760 ∼ 6.220) remained independent predictors of high-grade CS in the clinical model.

**Table 1:** Clinical factors of the training and validation sets

Clinical factors	Training set (*n* = 139)			Validation set (*n* = 57)	
	Low-grade CS (*n* = 80)	High-grade CS (*n* = 59)	*P*	Low-grade CS (*n* = 33)	High-grade CS (*n* = 24)
Gender (M/F)	35/45	32/27	0.300	15/18	11/13
Age, year	50.180 ± 1.490	55.410 ± 1.920	0.010	49.820 ± 2.270	55.880 ± 3.350
Tumor size (mm)	56.140 ± 3.540	74.220 ± 5.110	<0.001	53.850 ± 7.130	72.190 ± 9.870
Tumor location (long bone/non-long bone)	54/26	37/22	0.340	21/12	14/10
Tumor type (central/peripheral)	68/12	54/5	0.640	23/10	16/8
Depth of endosteal scalloping (Deep/Not deep)	33/47	46/13	<0.001	14/19	17/7
Entrapped fat (Yes/No)	6/74	1/58	0.090	2/31	0/24
Calcification (Yes/No)	76/4	50/9	0.100	30/3	22/2
Bone expansion (Yes/No)	53/27	47/12	0.310	21/12	13/11
Cortical destruction (Yes/No)	74/6	55/4	0.320	26/7	22/2
Active periostitis (Yes/No)	66/14	57/2	0.010	28/5	23/1
Soft tissue mass (Yes/No)	40/40	46/13	<0.001	17/16	18/6

M: male; F: female; CS: chondrosarcoma; *P*: the *P* value of comparison between low-grade CS and high-grade CS in training set; Numerical data are presented as mean ± standard deviation, categorical data as numbers (*n*)

### RS construction

**Fig. 3:**
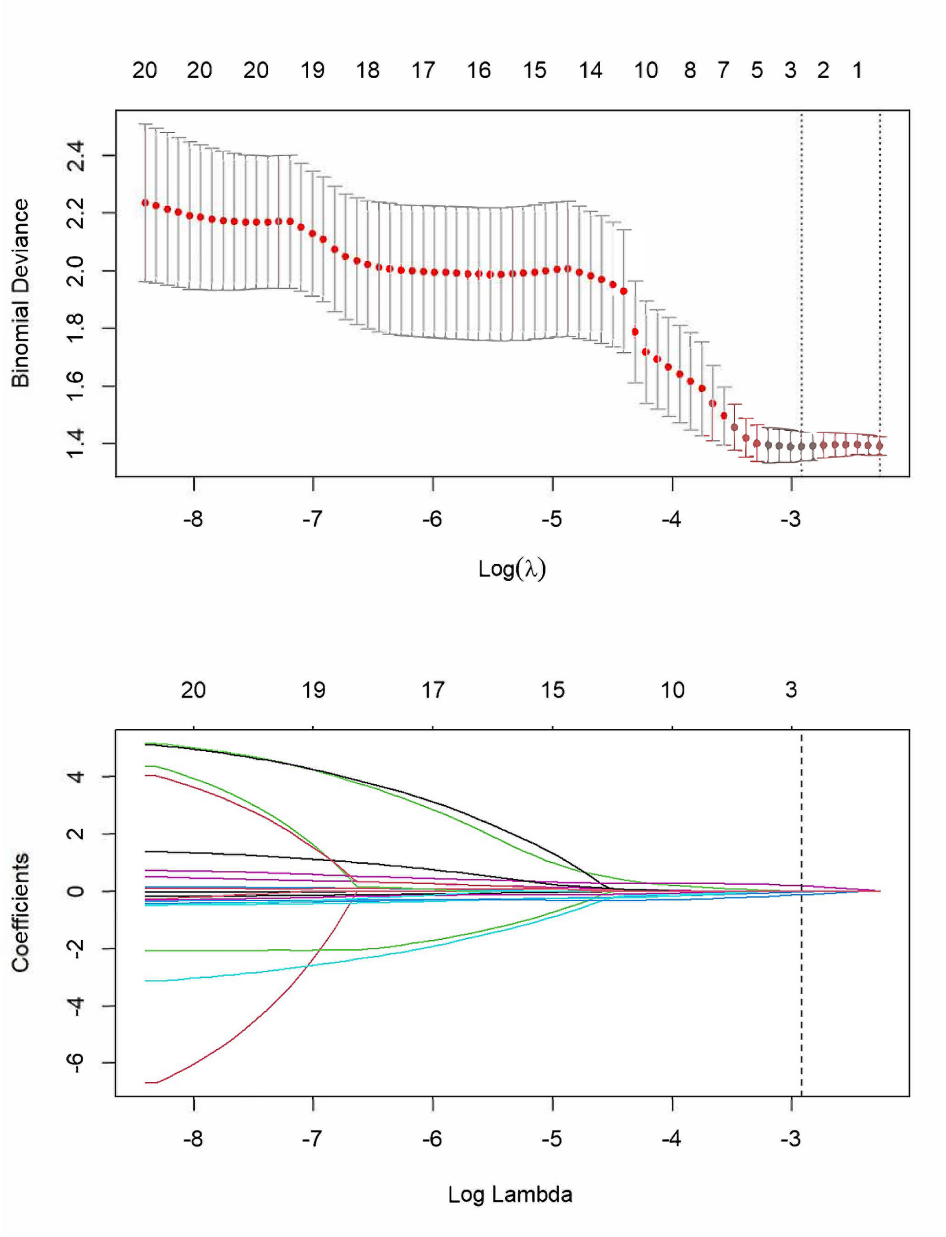
Radiomic-feature extraction using the least absolute shrinkage and selection operator (LASSO) regression model. **(a)** Tenfold cross-validation via the minimum error criterion was used for tuning parameter (λ) selection in LASSO. **(b)** LASSO coefficient profiles of the 1409 radiomic features. A coefficient-profile plot was generated versus the selected log λ value using tenfold cross-validation

Of the 1409 radiomics features, 1081 stable features were selected with ICC values greater than 0.750. Subsequently, 30 radiomics features were identified using ANOVA, indicating significant differences between low-grade and high-grade CS (*P* < 0.050). Next, the chosen 30 radiomics features were used as input to the LASSO Cox regression model to determine the most valuable factors for building the RS (Fig. 3). Finally, three radiomics features (Table 2) were selected to develop the RS. An optimal tuned regularization parameter under the minimum criteria was determined using 10-fold cross validation. The Rad-score formula was calculated via a linear combination of selected features and their respective weighted coefficients. Rad-score = -0.307 - A*0.014 - B*0.117 + C*0.193. The Rad-scores displayed significant differences between low-grade and high-grade CS in the training and validation cohorts (Table 3).

**Table 2:** Radiomics features selection results

Variables	Radiomics feature name
A	10 Percentile. First order. LBP-2D
B	Median. First order. Wavelet-HLL
C	Size Zone Non-uniformity. GLSZM. Wavelet-HLL

GLSZM: gray level size zone matrix;

**Table 3:** The results of Rad-score and Nomo-score in the training and validation sets

	Training set				Validation set		
	Low-grade CS	High-grade CS	P1		Low-grade CS	High-grade CS	P2
Rad-score	-0.361 ± 0.023	0.189 ± 0.073	< 0.001		-0.368 ± 0.042	0.289 ± 0.122	< 0.001
Nomo-score	0.111 ± 0.022	0.576 ± 0.052	< 0.001		0.106 ± 0.037	0.789 ± 0.115	< 0.001

Numerical data are presented as mean ± standard deviation

Rad-score: radiomics score; Nomo-score: nomogram score

CS: chondrosarcoma

### RN construction and validation of different models

**Fig. 4:**
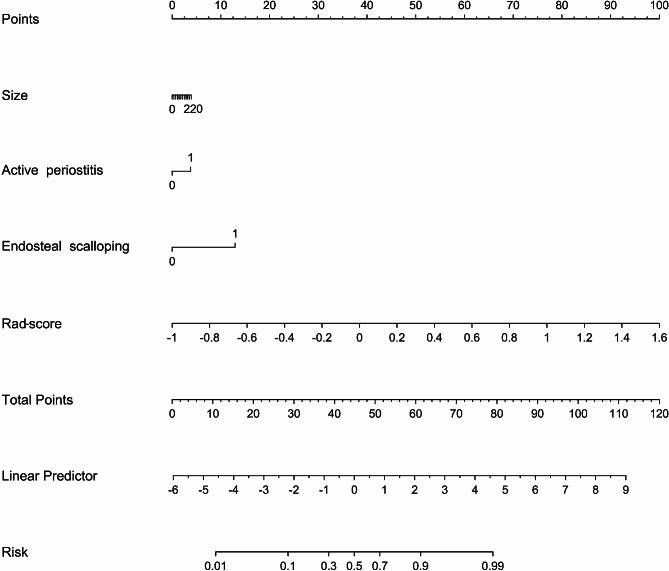
The radiomics nomogram (RN) of the training cohort incorporates valuable clinical factors and Rad-score. The clinical factors includes tumor size, active periostitis and endosteal scalloping

**Fig. 5:**
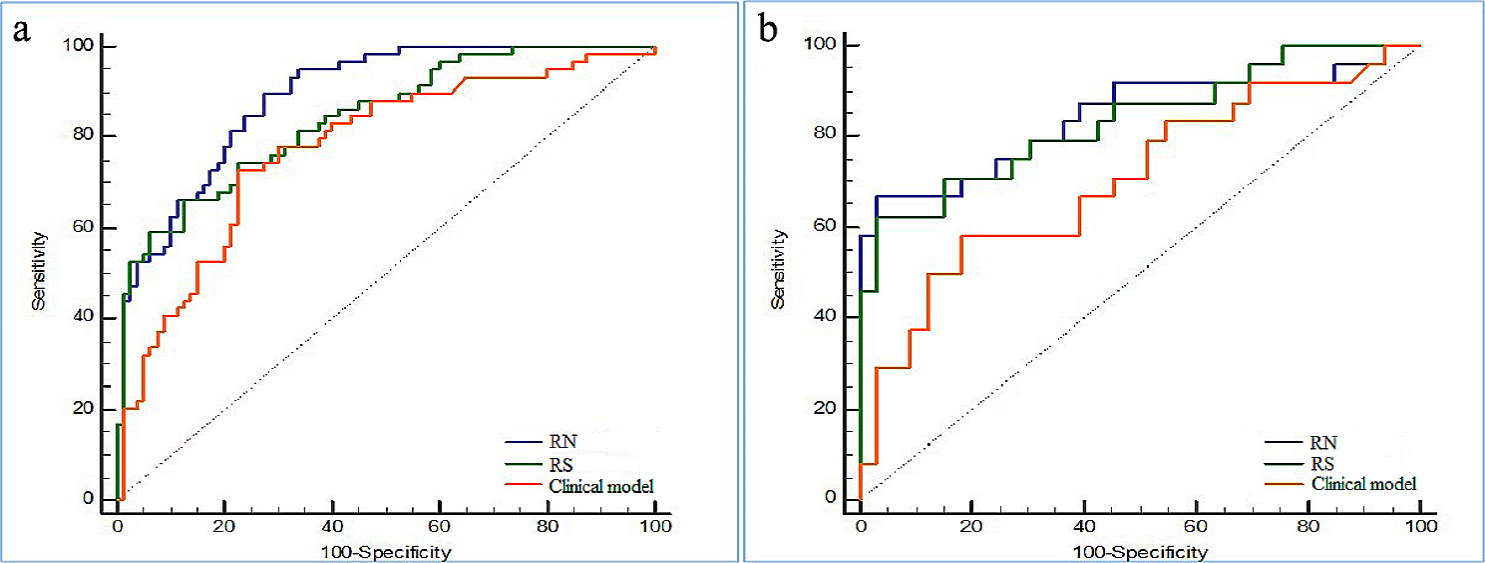
The receiver operating characteristic (ROC) curves for the clinical model, radiomics signature (RS) and radiomics nomogram (RN) in both the training **(a)** and validation sets **(b)**

Three selected clinical factors (size, endosteal scalloping and active periostitis) and Rad-score were combined to construct the RN (Fig. 4). Based on the above factors, the Nomo-score exhibited significant differences between low-grade and high-grade CS in both the training and validation cohorts (Table 3). The diagnostic performance of the three individual models is summarized in Table 4. Compared with the clinical model, both the RN and RS demonstrated superior discrimination in distinguishing low-grade and high-grade CS in both the training (*P* = 0.002, 0.020, respectively) and external-validation (*P* = 0.003, 0.040, respectively) cohorts (Fig. 5).

**Table 4:** Diagnostic performance of the clinical model, RS and RN

Model	AUC (95%CI)	Sensitivity %	Specificity %	Accuracy %
**Training set** (*n* = 139)				
Clinical model	0.776 (0.696–0.855)	72.900	76.300	74.800
RS	0.843 (0.778–0.908)	57.600	93.800	78.400
RN	0.890 (0.839–0.941)	64.400	88.800	78.400
**Validation set** (*n* = 57)				
Clinical model	0.705 (0.563–0.847)	58.300	75.800	68.400
RS	0.835 (0.724–0.945)	62.500	90.900	78.900
RN	0.842 (0.727–0.958)	66.700	90.900	80.700

CI: confidence interval; Data in the parentheses are raw data

RS: radiomics signature

RN: radiomics nomogram

### Follow‑up and Survival prediction

The medium follow-up period was 24 months (range: 1–155 months). The overall recurrence rate was 25% (49/196) up to the last follow-up. The median RFS was 13 months (range, 0–115 months) and 28 months (range, 0–155 months) in the recurrent and non-recurrent patients, respectively.

**Fig. 6:**
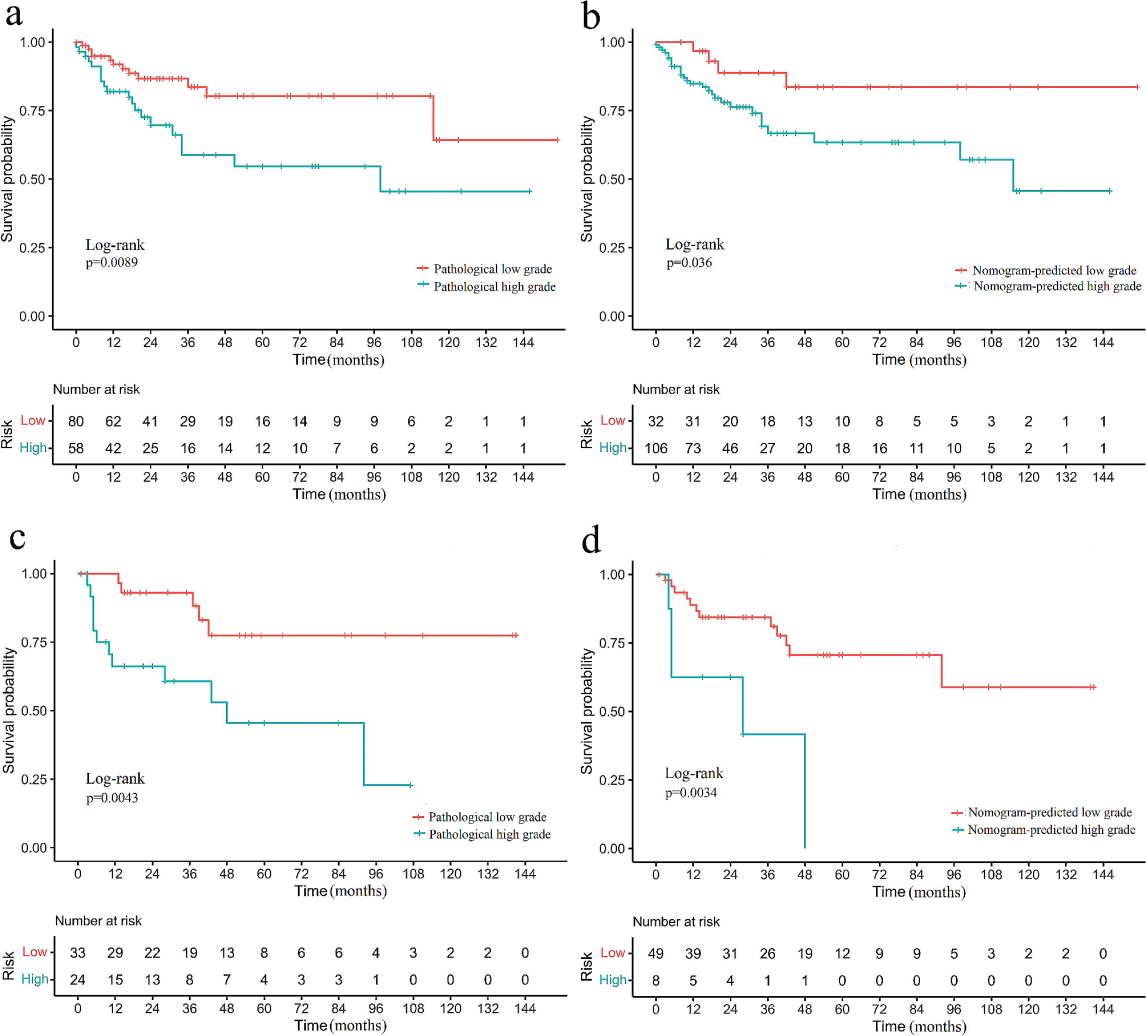
Kaplan-Meier estimate of recurrence-free survival (RFS) by dichotomized chondrosarcoma (CS) grade in patients with low-grade CS and high-grade CS. **(a)** Pathological grade in training group. **(b)** Nomogram-predicted grade in the training group; **(c)** Pathological grade in test group; **(d)** Nomogram-predicted grade in test group

The C-index for actual grade of CS with the RFS was 0.845 and 0.840 in the training and external-validation cohorts, respectively. The C-index for RN-predicted grade with the RFS was 0.813 and 0.810 in the training and external-validation cohorts, respectively. The optimal cut point of the Nomo-score was 0.410. The patients were dichotomized based on the cut point. A correlation between Nomo-score and RFS was found by the Kaplan-Meier survival analysis in both the training and test cohort (log-rank *P* < 0.050, Fig. 6). Patients with high Nomo-score tumors were 2.669 times more likely to suffer recurrence than those with low Nomo-score tumors (HR, 2.669; 95% CI, 0.988–7.208; *P* < 0.001).

## Discussion

Clinical decision-making ranges from wide resection or amputation to surveillance or curettage for high-grade to low-grade CS, respectively. However, due to the overlap of imaging features and low reliability, distinguishing these two entities with conventional modality is a challenge even among experienced radiologists and pathologists. In the present study, we constructed a CT-based RN for differentiating high-grade from low-grade CS using a non-invasive modality, and validated it in an external test cohort. The AUC of the RN in distinguishing high-grade from low-grade CS were 0.842 in the validation cohort. Additionally, the histologic grade stratified by the RN was found to be associated with RFS of CS patients.

In agreement with previous studies, size, endosteal scalloping and active periostitis were found to be the independent predictors for differentiating low-grade from high-grade CS. Fritz et al. reported 116 patients with cartilaginous bone neoplasms (including 26 with low-grade CS and 37 with high-grade CS) and found tumor size to be a differentiating feature for grading of cartilaginous neoplasms [16]. Sharif et al. discovered that deep endosteal scalloping and active periostitis were more common in patients with high-grade CS than in those with low-grade CS [6]. In the present study, the AUC of the clinical model in differentiating low-grade CS from high-grade CS was 0.705 in the validation cohort.

Radiomics, based on a large number of imaging characteristics, has been regarded as an effective modality to predict biological and histological features of tumors beyond visual assessment on CT/MR images. A few studies have suggested radiomics as a favorable diagnostic tool in preoperative classification of CS. Gitto et al. analyzed 158 cases of ACT and grade II CS, and demonstrated that an MRI radiomics-based machine learning had a high performance (AUC = 0.880) in distinguishing grade I CS (or ACT) from grade II CS of the long bones [15]. However, grade III CS was not included in the study. Gitto et al. also developed a CT radiomics-based machine learning for classification of ACT and higher-grade CS of long bones with 120 patients [14]. They found that a machine-learning classifier (LogitBoost) showed good accuracy in classifying the two entities with an AUC of 0.780 in the test cohort. Being different from these previous studies, Fritz et al. assessed the diagnostic accuracy of quantitative MRI-based texture analysis for grading cartilaginous bone tumors, and found that no significant independent texture predictors existed in differentiating low-grade from high-grade CS [16]. That maybe due to the relatively sample of the study, including 26 low-grade CSs and 37 high-grade CSs. Yin et al. showed that the clinical RN based on 3D Multi-parametric MRI features and clinical data had good performance in estimating early recurrence of pelvic CS, achieving an AUC of 0.891 in the validation set [13]. Among these studies regarding CS, few focused on the CT-based RN for predicting histologic grade and outcome of the CS.

In contrast with previous studies, several improvements need to be noted for the present study. First, 196 CS patients were comprised in our study from multicenter. To date, according to our knowledge, this is the largest population of study in differentiating high-grade CS from low-grade CS based on a RN. Besides, an independent external validation was also performed in the present study. Second, we combined the conventional imaging features and radiomics to detail the tumor heterogeneity based on CT images. Conventional imaging allows visible characterization extraction of tumor heterogeneity. Compared with conventional imaging, radiomics has been proven to be an effective imaging modality to identify histological and biological characteristics of tumors beyond visual assessment. Radiomics could extract and analyze an in-depth invisible quantitative features of tumor heterogeneity from images. In the present study, radiomics combined with conventional CT features showing a good performance in differentiating low-grade CS from high-grade CS (AUC = 0.891). Third, Previous studies identified several clinical factors to be related to the prognosis of CS patients, including grade, tumor size, tumor location, as well as resection margin [5, 20, 21]. In the present study, in addition to identifying the grade of CS, we also applied the histologic grade as a stratifying factor to evaluate the survival prediction in patients with CS. The RFS showed significant differences between the RN-predicted high-grade and low-grade groups. Furthermore, the RN achieved a high C-index in predicting RFS (0.810), indicating the prognostic value of the CT-based RN in the management of CS patients.

Notwithstanding, certain limitations of the present study need to be taken into account. First, due to the retrospective study nature, selection bias occurred potentially. Second, only non-contrast CT was used to build the RS. Advanced radiological modalities, including non-contrast MRI and dynamic contrast-enhanced CT/MRI, could be added in future studies to achieve higher-level evidence for distinguishing the two entities. Third, different CT scanners were included in the multicenter study. To standardize the CT images, several steps (image resampling, gray-level normalization and discretization) were performed before feature extraction. Finally, manually delineation of ROI was time-consuming. A deep learning modality for automatic segmentation of tumors was considered in the later studies.

## Conclusions

A CT-based RN provided favorable performance in preoperatively predicting histologic grade and outcome of CS. This could aid in facilitating individualized treatment plans for CS patients.

### Electronic supplementary material

Below is the link to the electronic supplementary material.

Supplementary Material 1

Supplementary Material 2

## Data Availability

Not applicable.

## Abbreviations

3D: Three-dimensional
ANOVA: Analysis of variance
AUC: Area under the receiver operator characteristic curve
CI: Confidence interval
CS: Chondrosarcoma
GLSZM: Gray-level size zone matrix
ICC: Inter-/intra-class correlation coefficient
LASSO: Least absolute shrinkage and selection operator
Nomo-score: Nomogram score
OR: Odds ratio
ROI: Regions of interest
ROC: Receiver operator characteristic
Rad-score: Radiomics score
RN: Radiomics nomogram
RS: Radiomics signature
RFS: Recurrence-free survival
