# Supplementary material for: A CT-based radiomics nomogram for predicting histologic grade and outcome in chondrosarcoma
Source: Cancer Imaging. 2024 Apr 11;24:50. doi: 10.1186/s40644-024-00695-7 (PMC11007871; doi:10.1186/s40644-024-00695-7)
Supplement: Supplementary file 1 — Supplementary Material 1 [file 40644_2024_695_MOESM1_ESM.docx]

**Supplementary Materials**

**CT scan protocols**

CT scan protocols of the 2 hospitals are shown in Table S1.

**Table S1 CT scan protocols**

| **CT scanner** | **CT 256** | **CT 128** | **CT 64** | **CT 64** | **CT 16** |
| --- | --- | --- | --- | --- | --- |
| Scanner model | Brilliance iCT 256 | Somatom Definition Flash | Somatom Sensation 64 | Discovery 750 | Aquilion One |
| Manufacturer | Philips | Siemens | Siemens | General Electric | Toshiba |
| Gantry rotation time (s) | 0.5 | 0.28 | 0.5 | 0.5 | 0.5 |
| Tube voltage (kV) | 120 | 120 | 120 | 120 | 120 |
| Tube current | 250 mA | Ref. 200 mAs (Care Dose 4D) | 200 mAs | 200-400 mA (automatic tube current modulation) | 100-400 mA |
| Detector collimation (mm) | 0.625 | 0.6 | 0.6 | 0.625 | 1 |
| Matrix | 512×512 | 512×512 | 512×512 | 512×512 | 512×512 |
| Pitch | 0.915 | 1.0 | 1.0 | 1.375 | 0.9375 |
| Slice thickness (mm) | 5 | 5 | 5 | 5 | 5 |
| Hospital | a | a | a | c | b |

Note: s (second); kV (kilovolt); mA (milliampere); mm (milimetre);

a. The Affiliated Hospital of Qingdao University;

b. The Affiliated Hospital of Shandong University of Traditional Chinese Medicine;

c. Shandong Provincial Hospital Affiliated to Shandong First Medical University.

**Segmentation of the region of interest (ROI)**

Three-dimensional (3-D) segmentation of ROI was performed using the ITK-SNAP software (Version 3.8, www.itksnap.org). Contouring was drawn within the borders of the tumors on CT images, avoiding covering the adjacent tissue and air.

**Radiomics features extraction**

There are four kinds of radiomics features: (1) 18 intensity statistic features, including features that quantitatively delineate the distribution of voxel intensities within the ROIs through commonly used and basic metrics; (2) 14 shape features, including 3-D features, which reflect the shape and size of the ROIs; (3) 93 texture features, which are composed of features calculated by gray level co-occurrence matrix (GLCM), gray level run length matrix (GLRLM), gray level size zone matrix (GLSZM), gray level dependence matrix (GLDM), and neighboring gray tone difference matrix (NGTDM) quantifying the heterogeneity differences of ROIs; and (4) 1284 filter and wavelet features, which include the intensity and texture features derived from filter transformation and wavelet transformation of the original images, obtained by applying filters such exponential, logarithm, square, square root, gradient, lbp_2D, lbp_3D_k and wavelet (wavelet-LHL, wavelet-LHH, wavelet-HLL, wavelet-LLH, wavelet-HLH, wavelet-HHH, wavelet-HHL and wavelet-LLL). The instructions of theradiomics features are shown in Pyradiomics document (Version 3.0) on https://pyradiomics.readthedocs.io.

The 3D segmentation of regions of interest (ROIs) was conducted by two musculoskeletal radiologists who were blinded to each other (Reader 1, L. XL, with 10 years’ experience; Reader 2, D. F, with 13 years’ experience) using the ITK-SNAP software (Version 3.8, www.itksnap.org). The axial sequence was preferred for image segmentation, with the coronal or sagittal sequence as an alternative. ROIs were manually segmented along the tumor borders slice-by-slice on CT images. Considering the different CT scanners used in our study, the following image preprocessing steps were followed using to ensure the conservation of scale: (1) image resampling (1×1×1 mm^3^ isotropic voxel using linear interpolation), and (2) grey level normalization and discretization (bins of 25 Hounsfield Units (HU) ^[1]^.

**References:**

[1] Gitto S, Cuocolo R, Annovazzi A, Anelli V, Acquasanta M, Cincotta A, Albano D, Chianca V, Ferraresi V, Messina C, Zoccali C, Armiraglio E, Parafioriti A, Sciuto R, Luzzati A, Biagini R, Imbriaco M, Sconfienza LM. CT radiomics-based machine learning classification of atypical cartilaginous tumours and appendicular chondrosarcomas. EBIOMEDICINE 2021;68:103407.
