# Supplementary material for: A CT-based radiomics nomogram for predicting histologic grade and outcome in chondrosarcoma
Source: Cancer Imaging. 2024 Apr 11;24:50. doi: 10.1186/s40644-024-00695-7 (PMC11007871; doi:10.1186/s40644-024-00695-7)
Supplement: Supplementary file 2 — Supplementary Material 2 [file 40644_2024_695_MOESM2_ESM.docx]

Dear reviewers,

Thank you for your E-mail for our manuscript entitled "A CT-based radiomics nomogram for predicting histologic grade and outcome in chondrosarcoma" (No. CAIG-D-23-00309). We have responded to the reviewer’s comment. Revised portion are marked in red in the paper. Point-by-point answers are as follows.

**Responses to Reviewer:**

1. I kindly suggest using the name of the methods, e.g. local binary pattern 2D, 3D and sub-channels of wavelets. in page 11 first paragraph. For the readers with different backgrounds, these terms will be confusing.

**[Answer]** Thank you for your comment. We have made revisions to clarify the name of the methods in page 11. The revisions were as follows:

In addition, fifteen filters were applied to the original images to derive specific images for each patient, including exponential, logarithm, square, square root, gradient, local binary patterns-two-dimension, local binary patterns-three-dimension-k, and wavelets [low-high-low (LHL), low-high-high (LHH), high-low-low (HLL), low-low-high (LLH), high-low-high (HLH), high-high-high (HHH), high-high-low (HHL), and low-low-low (LLL)].

Your comments and suggestions are really appreciated. Thank you.
